# What is the impact of the virome and mycobiome on female reproductive tract health? A systematic scoping review

**DOI:** 10.3389/fimmu.2026.1749584

**Published:** 2026-04-10

**Authors:** Ying Liu, Xingya Liao, Qimeng Chen, Hongyan Wang, Hualei Dai

**Affiliations:** 1 Department of Gynecology, The Third Affiliated Hospital of Kunming Medical University, Yunnan Cancer Hospital, Peking University Cancer Hospital, Kunming, Yunnan, China; 2 Cardiovascular Center, The Affiliated Hospital of Yunnan University, Yunnan University, Kunming, Yunnan, China; 3 School of Medicine, Yunnan University, Kunming, Yunnan, China

**Keywords:** bacterial vaginosis (BV), female reproductive tract microbiome, mycobiome, pan-microbiome, virome

## Abstract

**Background:**

Traditional research on the female reproductive tract (FRT) microbiome has focused on the dominance of bacteria, particularly *Lactobacillus*, as a marker of health. This bacteriocentric paradigm, however, cannot fully explain clinical enigmas like the high recurrence of bacterial vaginosis (BV) or the persistence of HPV infection. This review introduces a new pan-microbiome framework that highlights the overlooked roles of the virome and mycobiome as the ecosystem’s neglected components.

**Methods:**

We conducted a systematic scoping review following the PRISMA-ScR guidelines. We searched PubMed, Embase, and Web of Science databases for studies published up to October 2025. Inclusion criteria focused on original research and metagenomic studies examining the female reproductive tract (FRT) virome, mycobiome, and bacteriome, specifically their interactions and clinical associations with bacterial vaginosis (BV) and HPV persistence. Data were extracted and synthesized to evaluate the pan-microbiome framework.

**Results:**

The virome and mycobiome, despite their low biomass, are increasingly recognized as potential ecosystem modulators. Bacteriophages, for instance, are proposed to act as community “modulators,” either through lytic cycles that maintain bacterial diversity or lysogenic cycles that may contribute to stabilizing pathogenic biofilms in dysbiosis like BV by introducing virulence genes. Similarly, fungi like *Candida* can transition from harmless commensals to pathogens when the protective bacterial balance is disturbed.

**Conclusion:**

FRT health is an emergent property of the complex interactions among bacteria, viruses, and fungi. A comprehensive understanding requires a pan-microbiome perspective. Future therapeutic strategies should move beyond a “one-bug, one-drug” approach toward “ecosystem restoration,” using targeted methods like phage therapy or vaginal microbiota transplantation to attempt to restore the balance of the entire microbial community.

## 1 Introduction: transitioning from bacteriocentrism to a holistic ecosystem

Research on the female reproductive tract (FRT) microbiome has long been guided by a central tenet: a bacterial community dominated by *Lactobacillus* spp. with low species diversity is the hallmark of health (1). This bacteriocentric paradigm has profoundly shaped our understanding of FRT physiology and pathology, forming the cornerstone of clinical diagnostic and therapeutic strategies. According to this model, lactobacilli establish a robust defensive barrier by producing lactic acid to maintain an acidic vaginal environment, secreting antimicrobial compounds, and competitively excluding pathogens (2). When this equilibrium is disrupted—characterized by a depletion of lactobacilli and an overgrowth of diverse anaerobic bacteria—it leads to dysbiotic states such as bacterial vaginosis (BV), which increases the risk of adverse pregnancy outcomes, sexually transmitted infections (STIs), and gynecological diseases (3).

However, this seemingly elegant model falls short in explaining complex clinical phenomena. For instance, despite the temporary clearance of BV-associated bacteria with antibiotic treatment, recurrence rates are as high as 80% within nine months (4). Furthermore, bacterial profiling alone fails to provide a complete explanation for the inflammatory pathology of endometriosis or the individual variations in the persistence of human papillomavirus (HPV) infection (5). These clinical enigmas suggest the presence of critical regulatory forces within the FRT ecosystem that extend beyond bacteria and have been largely overlooked.

This review introduces a new conceptual framework that posits the FRT virome (including eukaryotic viruses and bacteriophages) and mycobiome as the functional “under-characterized components” of this ecosystem. While these components represent a smaller fraction of the total biomass compared to bacteria, emerging metagenomic evidence, analogous to findings in environmental ecosystems, suggests they may influence ecosystem structure and stability (6). By integrating viral and fungal dynamics, we propose a pan-microbiome framework to address the limitations of the bacteriocentric model.

This framework offers theoretical mechanistic insights into clinical challenges, such as the recalcitrance of BV biofilms and the variability of HPV clearance, positing that these outcomes may be emergent properties of multi-kingdom interactions rather than bacterial dynamics alone (7). Therefore, the objective of this scoping review is to map the available evidence on non-bacterial components of the FRT and, based on this synthesis, construct a conceptual model of pan-microbiome interactions (8). We systematically categorize the composition and potential regulatory functions of each microbial kingdom, explore their crosstalk, and discuss how this integrated perspective might generate new hypotheses for understanding key clinical diseases and guiding future therapeutic research.

## 2 Materials and methods

### 2.1 Search strategy and selection criteria

This study was designed as a systematic scoping review to map the available evidence regarding the “pan-microbiome” framework within the female reproductive tract (FRT). The review was conducted and reported in strict accordance with the Preferred Reporting Items for Systematic Reviews and Meta-Analyses extension for Scoping Reviews (PRISMA-ScR) guidelines.

### 2.2 Search strategy

A comprehensive systematic search was conducted in PubMed, Embase, and Web of Science databases for articles published from inception to October 2025. The search strategy combined controlled vocabulary (MeSH terms) and free-text keywords relevant to the research question, including: “female reproductive tract,” “vaginal microbiota,” “virome,” “mycobiome,” “bacteriophage,” “Candida,” “bacterial vaginosis,” and “human papillomavirus.” To ensure literature saturation, the reference lists of identified key reviews and included primary studies were manually screened for additional relevant records.

### 2.3 Eligibility criteria

Studies were selected based on the following inclusion criteria (1): original research articles, metagenomic analyses, or longitudinal studies (2); studies investigating the composition, function, or interactions of the FRT virome (including bacteriophages) and mycobiome, specifically in the context of their relationship with the bacteriome (3); studies reporting clinical outcomes associated with dysbiosis, such as Bacterial Vaginosis (BV), HPV persistence, or reproductive health complications; and (4) articles published in peer-reviewed journals in English. Exclusion criteria were (1): editorials, commentaries, and conference abstracts (2); studies focusing solely on single-pathogen infections without an ecological or microbiome-interaction perspective (3); studies with insufficient data on microbial interactions; and (4) non-English publications.

### 2.4 Study selection and data extraction

**Figure 1:**
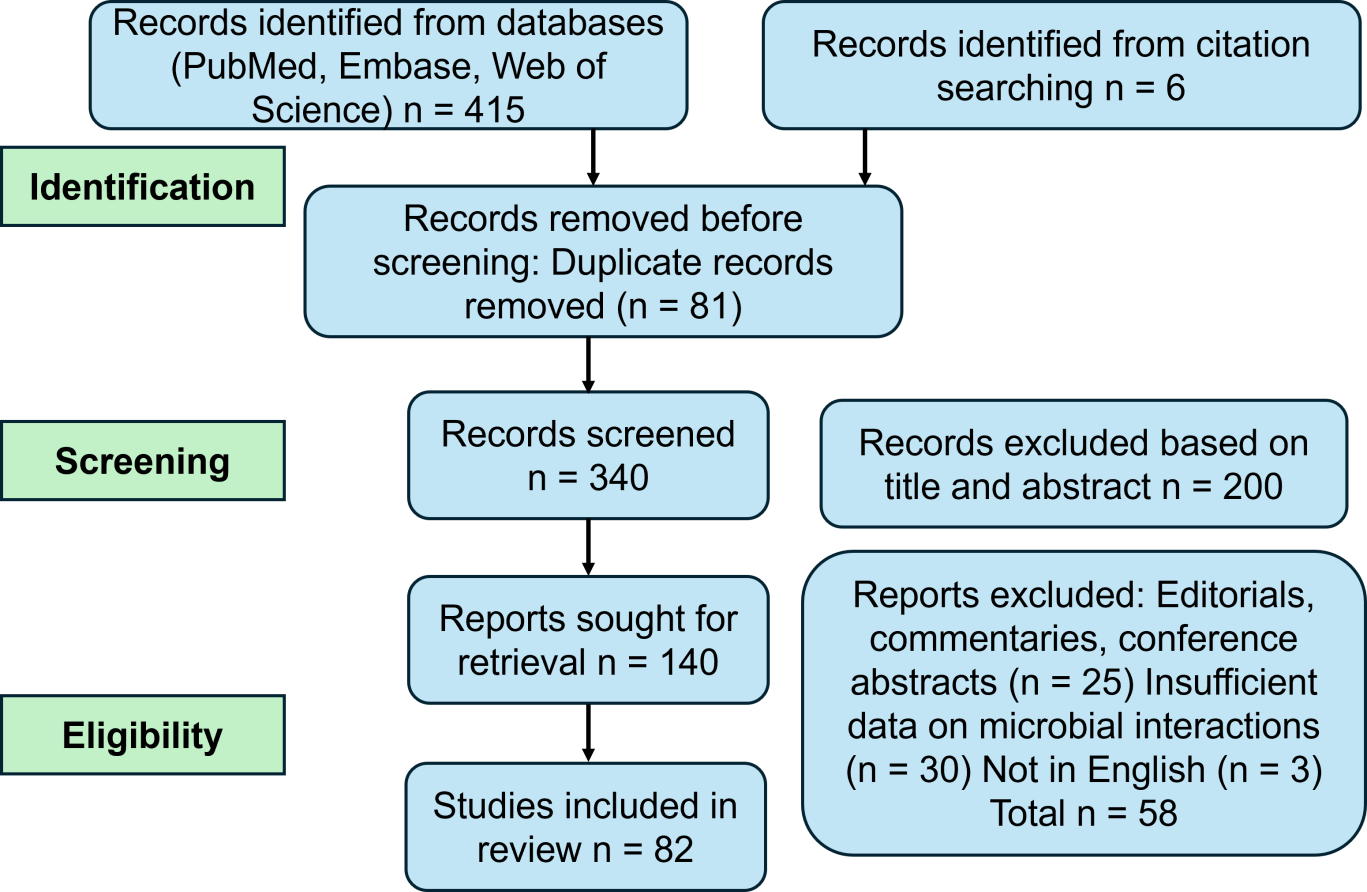
PRISMA.

The selection process followed the PRISMA flow diagram (**Figure 1**). Initial database searching identified 415 records, and citation searching identified an additional 6 records. After removing 81 duplicates, 340 records were screened by title and abstract. Of these, 140 full-text reports were sought for retrieval and eligibility assessment. Two independent reviewers (Y.L. and Q.C.) performed the screening and data extraction. Disagreements were resolved through consensus or consultation with a third reviewer (H.D.). A total of 58 reports were excluded for reasons including: being editorials/commentaries/abstracts (n = 25), lacking sufficient data on microbial interactions (n = 30), or being unavailable in English (n = 3). Finally, 82 studies met the inclusion criteria and were included in this review (**Table 1**).

**Table 1:** Summary of literature search and study selection process.

Screening stage	Description	Count (n)
Identification	Records identified from database searching (PubMed, Embase, Web of Science)	415
	Additional records identified through citation searching	6
	Total records identified	421
Screening	Records after duplicates removed (n = 81 removed)	340
	Records excluded at title/abstract level	(200)
Eligibility	Full-text articles assessed for eligibility	140
	Full-text articles excluded	58
	Reasons for exclusion:	
	1. Editorials, commentaries, and conference abstracts	25
	2. Insufficient data on microbial interactions	30
	3. Non-English publications	3
Inclusion	Studies included in the systematic scoping review	82

### 2.5 Data synthesis

Data were extracted regarding study design, population characteristics, microbial domains analyzed (bacteria, viruses, fungi), and key findings related to cross-kingdom interactions. Given the significant heterogeneity in study designs (ranging from cross-sectional 16S rRNA surveys to shotgun metagenomics) and the diverse clinical outcomes reported, a quantitative meta-analysis was not feasible. Therefore, a narrative synthesis approach was adopted. Evidence was synthesized thematically to construct the “pan-microbiome” framework, categorizing findings into bacterial foundation, viral regulation, fungal opportunism, and their collective clinical implications.

## 3 Results

### 3.1 The bacterial foundation: community structure and function in homeostasis and dysbiosis

Unlike microbial ecosystems in other body sites, the health of the FRT is uniquely characterized by low species richness and the dominance of Lactobacillus species. This “ecological anomaly” relies on lactobacilli to metabolize glycogen into L-lactic acid, maintaining a vaginal pH below 4.5 and producing antimicrobial peptides that competitively exclude pathogens (9–13). However, “health” is not a monolithic state but a gradient of stability dependent on the specific Lactobacillus species present. Evidence indicates a functional hierarchy: Lactobacillus crispatus is associated with the most stable, “robust health,” whereas Lactobacillus iners, with its limited metabolic capacity and production of D-lactic acid, represents a “transitional” or permissive state more susceptible to disruption (9, 14–16).

To categorize these variations, the field has adopted the Community State Types (CSTs) framework proposed by Ravel et al (17). CSTs I, II, III, and V are dominated by L. crispatus, L. gasseri, L. iners, and L. jensenii, respectively. In contrast, CST IV represents a dysbiotic state characterized by a depletion of lactobacilli and high diversity of strict anaerobes (18–20). This shift is the hallmark of Bacterial Vaginosis (BV), where a polymicrobial biofilm formed by Gardnerella, Atopobium, and Prevotella replaces the protective Lactobacillus barrier. Distinct from BV, Aerobic Vaginitis (AV) involves aerobic pathogens like E. coli and triggers a pronounced host inflammatory response (21, 22). Understanding these bacterial foundations is rigorous, yet bacterial profiling alone often fails to explain recurrence and host variability, necessitating an examination of the broader pan-microbiome.

### 3.2 Unveiling the virome: viruses as regulators and residents

Metagenomic advances have revealed that the FRT virome is dominated not by eukaryotic viruses, but by bacteriophages, which constitute over 95% of the viral community (23). The vaginal virome is highly individual-specific and exhibits distinct signatures associated with bacterial CSTs. In healthy, L. crispatus-dominated states, the virome is primarily composed of Lactobacillus-specific phages (24). Conversely, in BV, there is a marked restructuring characterized by an expanded diversity of phages infecting anaerobes like Gardnerella.

Based on these compositional shifts, we posit a conceptual model where bacteriophages may act as “ecosystem-modulating components” of bacterial community structure through two primary mechanisms. First, lytic phages likely exert top-down control on bacterial populations, potentially maintaining diversity by preventing the overgrowth of specific strains (25). Second, and more critically for disease pathogenesis in contributing to the stability of dysbiosis. The enrichment of lysogenic sequences in BV samples supports the hypothesis that prophages may serve as genetic reservoirs (26). Drawing upon biological plausibility from non-FRT bacterial models (27, 28), these prophages could theoretically confer fitness advantages. While longitudinal functional validation is still required, this “phage-stabilized biofilm” model offers a plausible biological explanation for the high recurrence rates of BV that strictly bacterial models fail to fully elucidate.

### 3.3 The mycobiome: a kingdom of commensalism and opportunism

The mycobiome constitutes a low-biomass but functionally significant component of the FRT. In asymptomatic women, Candida albicans frequently exists as a commensal yeast, suggesting that a balanced mycobiome is compatible with health (29). The transition to Vulvovaginal Candidiasis (VVC) is driven by the morphological switch from yeast to invasive hyphae, accompanied by the secretion of virulence factors like candidalysin and biofilm formation (30, 31).

**Figure 2:**
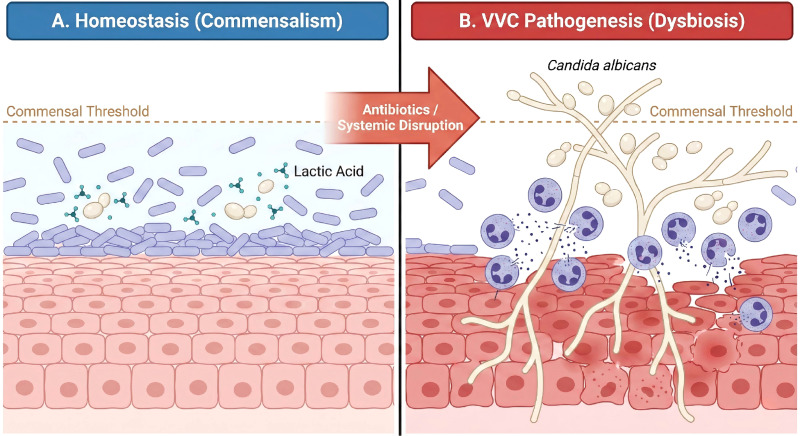
Conceptual model of the pathogenic switch of *Candida albicans* from commensalism to dysbiosis. **(A)** In a homeostatic environment (left), Lactobacillus species dominate the niche, producing lactic acid that maintains C. albicans in its varying yeast form and below a specific “Commensal Threshold.” **(B)** Following systemic disruption (e.g., broad-spectrum antibiotics), the depletion of lactobacilli creates a permissive environment. In this dysbiotic state (right), C. albicans proliferates beyond the threshold and undergoes a morphological transition to the invasive hyphal form. These hyphae penetrate the vaginal epithelium, triggering an influx of neutrophils and inflammatory damage, characterizing Vulvovaginal Candidiasis (VVC).

Recurrent VVC (RVVC) challenges the traditional view of simple infection, pointing instead to a breakdown in ecosystem regulation. We propose that clinical symptoms arise when the fungal burden exceeds a “commensal threshold” defined by the host-microbiome interaction (32) (**Figure 2**). In a healthy state, bacterial suppression and appropriate host immune surveillance likely maintain fungal levels below this threshold. Dysbiosis arises not necessarily from the introduction of a new pathogen, but from a failure of these regulatory networks—such as the loss of Lactobacillus-mediated inhibition or an aberrant immunopathological response by the host (33). This perspective shifts the focus from solely eradicating the fungus to restoring the homeostatic capacity of the FRT ecosystem.

### 3.4 Pan-microbiome synergies: complex interactions of cross-kingdom crosstalk

The health of the FRT is not determined by any single microbial kingdom in isolation but is an emergent property of the complex interactions among bacteria, viruses, and fungi. Understanding this cross-kingdom crosstalk is key to building a pan-microbiome perspective.

#### 3.4.1 Bacteria-fungus interactions: the *Lactobacillus-Candida* axis

In the FRT ecosystem, the relationship between lactobacilli and *Candida* is primarily antagonistic. This antagonism is achieved through several mechanisms. First, lactic acid and other organic acids produced by lactobacilli significantly inhibit the growth of *Candida*. More importantly, they effectively block the transition of *Candida* from its commensal yeast form to its pathogenic hyphal form, a critical step in preventing VVC (34). Second, lactobacilli compete with *Candida* for adhesion sites and nutrients, limiting its colonization on the vaginal epithelium (35).

However, a seemingly paradoxical observation from cross-sectional studies is that *Candida* colonization is positively correlated with *Lactobacillus* dominance and negatively correlated with BV-associated bacteria (36). This suggests that the acidic, healthy environment created by lactobacilli, while suppressing.

*Candida*’s pathogenicity, provides a suitable niche for its commensal colonization. Conversely, the high pH and anaerobic environment of BV are inhibitory to both *Candida* and lactobacilli. This finding reveals the complexity of bacteria-fungus interactions: lactobacilli appear to act as “regulators” rather than “eliminators.” Mechanistically, lactobacilli compete for adhesion sites and carbon sources while maintaining an acidic pH that restricts the yeast-to-hyphae transition—the primary virulence trait of Candida (37). This “colonization resistance” allows the host to tolerate low-abundance yeast forms as commensals while preventing the pathogenic tissue invasion associated with dysbiosis.

This dynamic equilibrium has important clinical implications. The development of VVC following antibiotic treatment for BV is a common phenomenon (38). The underlying mechanism is not that BV bacteria actively suppress *Candida*, but that antibiotics (like metronidazole) launch an “indiscriminate attack” on the entire bacterial community (both pathogenic anaerobes and residual beneficial lactobacilli), creating a “microbial vacuum.” As a eukaryote, *Candida* is unaffected by bacterial antibiotics and can rapidly proliferate in the absence of competition, breaching the pathogenic threshold and triggering symptomatic VVC. This example vividly illustrates that the stability of the mycobiome is highly dependent on the presence of a structurally intact bacterial community, whether healthy or dysbiotic.

#### 3.4.2 Virus-bacteria interactions: phage-driven ecosystem stability

As previously discussed, bacteriophages are a major driving force behind the dynamic changes in the FRT bacterial community. Through lytic predation, phages prevent the overgrowth of any single bacterial species, thereby maintaining community diversity. Through lysogenic integration, phages introduce new functional traits into the bacterial pangenome, accelerating their adaptive evolution. In dysbiotic states, phages potentially act as “contributors” in disease by promoting the stability of the BV biofilm.

#### 3.4.3 A conceptual model of three-kingdom dynamics

Based on the above, we can construct a three-kingdom dynamic conceptual model of the FRT ecosystem:

##### 3.4.3.1 Healthy homeostasis

**Figure 3:**
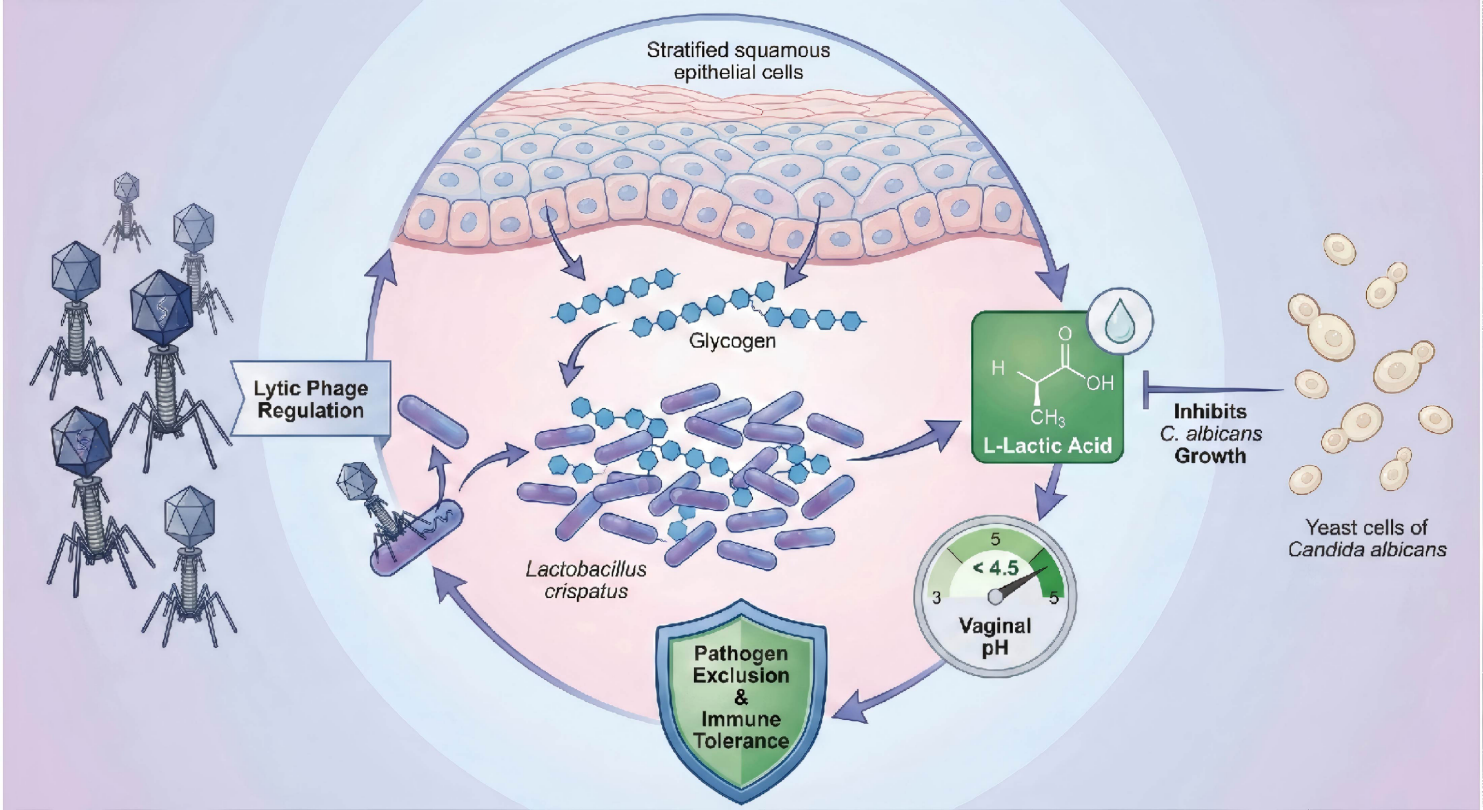
Schematic dynamics of a healthy female reproductive tract ecosystem. This diagram illustrates the cyclical maintenance of homeostasis. Vaginal epithelial cells release glycogen, which is metabolized by Lactobacillus crispatus into L-lactic acid, maintaining a protective pH < 4.5. This acidic environment inhibits the overgrowth of Candida albicans (keeping it in a commensal state) and excludes pathogens. The stability of this bacterial community is presumably modulated by lytic bacteriophages (“Lytic Phage Regulation”), which prevent the dominance of any single bacterial strain through top-down predator-prey dynamics.

A robust bacteriome dominated by *L. crispatus* maintains a strongly acidic environment by producing lactic acid. This bacterial community is itself dynamically regulated by its specific phages, preventing it from becoming overly monolithic. This acidic environment suppresses the pathogenic transition of *Candida* and inhibits the growth of anaerobic bacteria. A dynamic equilibrium is achieved among the three kingdoms, collectively maintaining FRT health (**Figure 3**).

##### 3.4.3.2 Dysbiotic state (e.g., BV)

**Figure 4:**
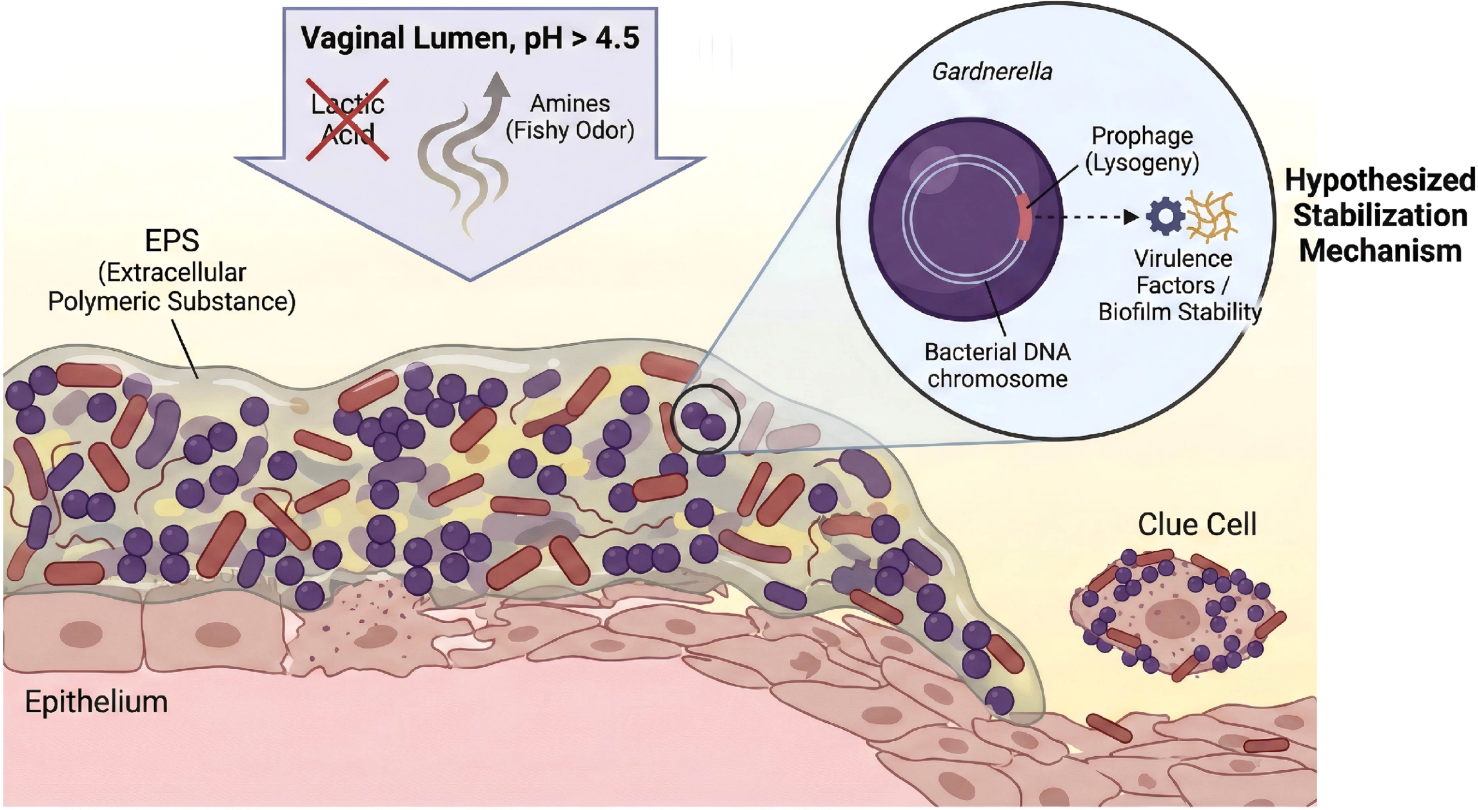
Hypothesized architecture of a dysbiotic biofilm in bacterial vaginosis. The main panel depicts the vaginal epithelium covered by a polymicrobial biofilm embedded in Extracellular Polymeric Substance (EPS), characteristic of BV (pH > 4.5). The exfoliation of epithelial cells coated in bacteria forms “Clue Cells,” a diagnostic hallmark. The inset (circle) details the hypothesized stabilization mechanism: Lysogenic prophages integrated into the chromosomes of anaerobes (e.g., Gardnerella) may encode virulence factors or biofilm-stability proteins. This phage-bacteria interaction is proposed to enhance the persistence of the biofilm against host immunity and antibiotics. This schematic represents a conceptual synthesis of multi-omics associations and is intended for illustrative purposes, acknowledging that *in vivo* interactions are likely heterogeneous.

When the *Lactobacillus* community collapses for various reasons (e.g., antibiotics, hormonal fluctuations, or perhaps a lytic phage outbreak), the ecosystem enters a state of dysbiosis. The anaerobic bacterial community begins to expand and rapidly forms a polymicrobial biofilm that is stabilized and fortified by lysogenic phages. The high-pH, anaerobic environment created by this biofilm further inhibits the survival of lactobacilli and commensal fungi, making the dysbiotic state self-sustaining and leading to clinical disease (**Figure 4**).

The table below summarizes the key features of the three microbial kingdoms in the FRT under different conditions (**Table 2**).

**Table 2:** Characteristics of the three microbial kingdoms in the female reproductive tract.

Feature	Bacteriome	Virome	Mycobiome
Dominant Taxa (Health)	*Lactobacillus crispatus*	*Lactobacillus* phages (*Siphoviridae*)	*Candida albicans* (yeast form)
Dominant Taxa (Dysbiosis)	*Gardnerella*, *Prevotella*	Anaerobe phages (lysogenic)	Variable, often decreased abundance
Typical Diversity	Low	Medium to High	Low
Relative Abundance	High	Low	Very Low
Primary Role in Homeostasis	Acidification, barrier function	Top-down bacterial regulation	Commensal tolerance
Primary Role in Pathology	Biofilm formation, inflammation	Stabilization of dysbiosis, gene transfer	Morphological transition, immunopathology

### 3.5 Clinical implications of the pan-microbiome framework

Applying the pan-microbiome framework to specific clinical problems provides deeper insights into the onset, progression, and resolution of diseases.

#### 3.5.1 Reframing bacterial vaginosis as a multi-kingdom disease

BV should no longer be considered a purely bacterial disease. It is a complex, biofilm-based pathological state in which members of multiple kingdoms participate and which they collectively sustain. At its core is a dysbiotic bacteriome, but this dysbiotic community may be stabilized and structurally supported by a specific phageome. By endowing their bacterial hosts with enhanced survival and pathogenic capabilities, lysogenic phages are a key factor in the recalcitrance and high recurrence rate of BV biofilms. Future therapeutic strategies for BV must consider how to dismantle this bacteria-phage symbiosis, for example, through phage therapy or endolysins targeting key bacteria (like *Gardnerella*), to have a chance of breaking the vicious cycle of recurrence.

#### 3.5.2 HPV persistence and cervical carcinogenesis—a pan-microbiome cascade

**Figure 5:**
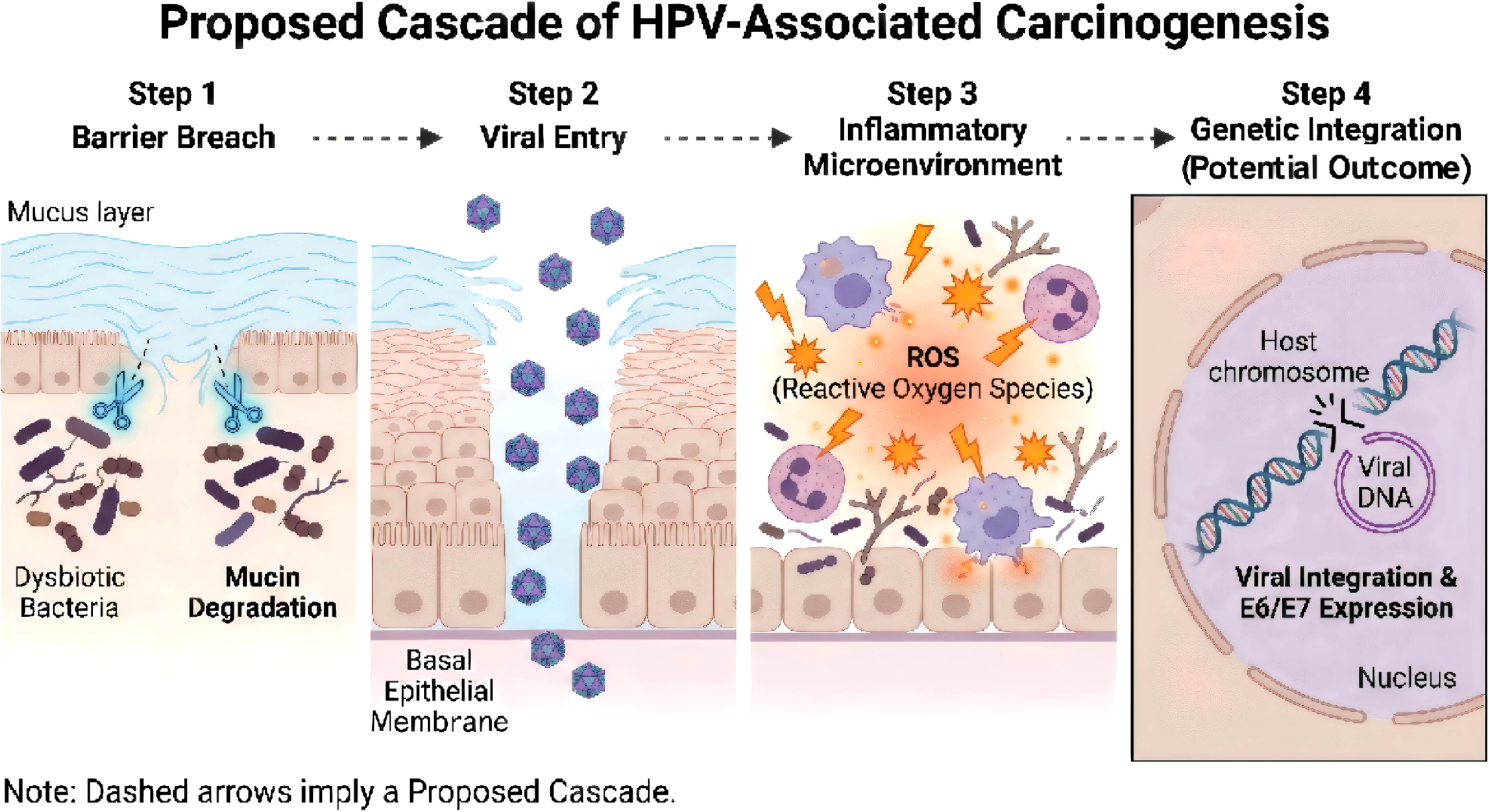
Proposed pan-microbiome cascade of HPV-associated carcinogenesis. This flowchart outlines a sequential mechanism linking dysbiosis to viral progression. (Step 1) Dysbiotic bacteria secrete hydrolytic enzymes (depicted as scissors) that degrade the protective mucin layer. (Step 2) This barrier breach facilitates the entry of HPV particles to the basal epithelial membrane. (Step 3) The persistent dysbiotic environment triggers chronic inflammation and the release of Reactive Oxygen Species (ROS) from immune cells. (Step 4) ROS-induced DNA damage promotes the integration of the HPV genome into the host chromosome, leading to uncontrolled E6/E7 oncogene expression. Note: Dashed arrows indicate that this pathway represents a conceptual synthesis of multi-omics associations rather than a strictly linear biological inevitability. The proposed outcomes are hypothesized based on current cross-sectional data and may vary significantly depending on host immune status and environmental co-factors.

The progression of HPV infection is strongly influenced by the local microenvironment (**Table 3**). We propose a hypothesized multi-step mechanistic cascade linking pan-microbiome dysbiosis to cervical carcinogenesis (**Figure 5**), although we note this model is synthesized primarily from cross-sectional associations and likely involves non-linear, host-dependent pathways. In this framework, a CST IV-like dysbiotic community initiates the process by producing hydrolytic enzymes (sialidases) that degrade the protective cervical mucus barrier, facilitating viral access to basal cells (39). Concurrently, bacterial metabolites induce chronic inflammation and oxidative stress (ROS), which may cause host DNA damage and promote viral genome integration (40). Furthermore, the specific immune milieu created by dysbiosis often favors immune tolerance rather than viral clearance. While the specific contribution of the mycobiome requires further elucidation, this integrated model suggests that restoring a L. crispatus-dominant state could theoretically interrupt this pathogenic cascade at multiple checkpoints.

**Table 3:** Microbial features and mechanisms associated with HPV persistence and cervical carcinogenesis.

Microbial feature	Associated outcome	Proposed mechanism
*Lactobacillus crispatus* dominance (CST I)	Viral clearance	Lactic acid production, maintenance of barrier integrity, modulation of antiviral immunity
*Lactobacillus iners* dominance (CST III)	Increased risk/Transitional state	Weaker acidification; production of inerolysin may facilitate viral entry
High-diversity community (CST IV)	Persistence/Progression	Chronic inflammation, reactive oxygen species (ROS) production, mucosal barrier disruption
*Sneathia* enrichment	Persistence/Progression	Induction of pro-inflammatory cytokines, disruption of epithelial barrier
*Gardnerella*/*Prevotella* enrichment	Persistence/Progression	Biofilm formation, metabolites promote inflammation, provide nutrients for other anaerobes
*Fusobacterium* enrichment	Progression to cancer	Production of genotoxic metabolites, suppression of anti-tumor immunity

#### 3.5.3 Endometriosis, infertility, and preterm birth

Dysbiosis of the pan-microbiome is also linked to other significant obstetrical and gynecological conditions. Emerging evidence suggests that both the FRT and gut microbiomes are dysbiotic in patients with endometriosis, and this dysbiosis may contribute to the disease’s development by modulating estrogen metabolism and inducing chronic inflammation (41). Similarly, the composition of the FRT microbiome, including microbial colonization of the upper reproductive tract (uterus, fallopian tubes), is thought to have a significant impact on fertility and the success rates of assisted reproductive technologies (42). During pregnancy, vaginal microbiome dysbiosis, particularly the absence of lactobacilli, is a key risk factor for adverse pregnancy outcomes such as preterm birth (43).

### 3.6 Therapeutic implications: from antibiotics to ecosystem modulation

The limitations of standard antibiotic regimens, particularly high recurrence rates, have spurred interest in strategies aimed at ecosystem restoration rather than simple pathogen eradication. Vaginal Microbiota Transplantation (VMT) attempts to re-establish a protective L. crispatus community, though its clinical application faces significant hurdles regarding donor screening and the long-term stability of the engrafted microbiome (44, 45).

More targeted experimental approaches include phage therapy and the use of endolysins. Unlike broad-spectrum antibiotics, these agents offer notable potential in preclinical models for precision editing of the microbiome—for instance, targeting Gardnerella within a biofilm while sparing beneficial lactobacilli (46). Endolysins, which degrade bacterial cell walls, have shown promise in preclinical models for disrupting biofilms, theoretically addressing a root cause of recurrence. However, the translation of these strategies to clinical practice requires rigorous validation of safety and efficacy in diverse populations, alongside the development of regulatory frameworks to address the complexity of introducing biological agents into the FRT environment.

### 3.7 Challenges and future directions

Translating the pan-microbiome vision into clinical reality requires overcoming significant challenges, primarily bridging the methodological gaps that currently obscure the non-bacterial components. A major bottleneck lies in sample processing, where standard extraction protocols optimized for bacteria often fail to lyse the robust cell walls of fungi or lose viral particles, leading to an underestimation of these kingdoms (47).

Furthermore, the reliance on amplicon-based sequencing (16S rRNA, ITS) creates taxonomic blind spots due to primer bias. While shotgun metagenomics offers a comprehensive alternative, it demands high sequencing depth to capture low-biomass viral and fungal signals against a high-background of host DNA (48). Compounding this is the scarcity of annotated reference genomes for vaginal viruses and fungi, which leaves a significant portion of sequencing reads as unclassified “microbial dark matter” (49).

Future research must therefore move beyond compositional descriptions to a functional understanding by integrating multi-omics layers. Metatranscriptomics and metaproteomics are essential to distinguish active microbial players from dormant ones and to identify the actual effector proteins driving ecosystem function. Additionally, metabolomics can reveal the chemical dialogue between the microbiome and the host, providing a direct readout of physiological states (50).Crucially, these multi-omics data must be anchored in well-designed longitudinal studies. Cross-sectional snapshots are insufficient to capture the dynamic temporal shifts of the FRT ecosystem driven by the menstrual cycle, pregnancy, or life events (51). Only longitudinal tracking can disentangle correlation from causation and identify the specific tipping points that lead to dysbiosis.

Finally, the integration of such high-dimensional, multi-kingdom data necessitates the application of advanced artificial intelligence (AI) and machine learning (ML) specifically tailored for microbiome interactions. Computational tools are critical for identifying non-linear patterns, such as virus-host interaction networks, that traditional statistics miss (52). However, the application of AI in this context requires rigor; algorithmic models trained on databases lacking diversity—such as those underrepresenting specific ethnicities or geographic regions—risk perpetuating biases in risk assessment (53, 54). Therefore, the development of predictive pan-microbiome models must prioritize validation across diverse populations to ensure equitable clinical utility (55). The future of the field depends not just on generating more data, but on developing sophisticated, bias-aware computational frameworks to interpret the FRT ecosystem as a functional whole.

## 4 Discussion

This review has systematically argued the limitations of the traditional bacteriocentric model and highlighted the necessity of viewing the virome and mycobiome as integral modulators of the FRT ecosystem. By uncovering the regulatoryroles of these microbial “under-characterized components” and their complex interactions with the bacteriome, we can gain a much deeper understanding of FRT health and disease.

An integrated, pan-microbiome perspective is providing a more comprehensive perspective in the field of women’s reproductive health. Future gynecological diagnostics may evolve from simple bacterial identification to comprehensive pan-microbiome risk assessments. Therapeutic approaches will also shift from broad-spectrum eradication strategies to ecological engineering aimed at precisely modulating and restoring the entire microbial ecosystem, such as phage therapy, endolysins, and VMT.

By embracing the complexity of the FRT pan-microbiome and striving to illuminate its non-bacterial elements, we will undoubtedly pave new ways for developing personalized medical solutions, ultimately improving the reproductive health and overall well-being of women worldwide.
